# Tackling Refractory Metastatic Colorectal Cancer: Future Perspectives

**DOI:** 10.3390/cancers13184506

**Published:** 2021-09-07

**Authors:** Nicola Personeni, Valeria Smiroldo, Emilio Francesco Giunta, Maria Giuseppina Prete, Lorenza Rimassa, Giacomo Bregni, Francesco Sclafani

**Affiliations:** 1 Department of Biomedical Sciences, Humanitas University, Via Rita Levi Montalcini 4, 20072 Milan, Italy; nicola.personeni@hunimed.eu (N.P.); maria.prete@cancercenter.humanitas.it (M.G.P.); 2 Medical Oncology and Hematology Unit, Humanitas Cancer Center, IRCCS Humanitas Research Hospital, Via Manzoni 56, 20089 Milan, Italy; valeria.smiroldo@cancercenter.humanitas.it; 3 Medical Oncology Department of Precision Medicine, University of Campania “Luigi Vanvitelli”, Via Pansini 5, 80131 Naples, Italy; emiliofrancesco.giunta@unicampania.it; 4 Department of Medical Oncology, Institut Jules Bordet–Université Libre de Bruxelles (ULB), Boulevard de Waterloo 121, 1000 Bruxelles, Belgium; Giacomo.Bregni@bordet.be (G.B.); francesco.sclafani@bordet.be (F.S.)

**Keywords:** colorectal cancer, metastatic, refractory, molecular characterization, biomarker-driven strategies, targeted agents

## Abstract

**Simple Summary:**

Metastatic colorectal cancer (mCRC) accounts for relevant cancer-related morbidity and mortality. Novel investigations have reshaped the molecular makeup of mCRC, emphasizing a high degree of heterogeneity that can be leveraged to establish a new concept of biomarker-guided therapy. In contrast to the old-fashioned, “one-size-fits-all” therapeutic approach, within a precision oncology approach, a deeper molecular selection is indeed felt to improve the efficacy of targeted systemic treatments. Here, we review available treatment options in patients with refractory mCRC, who have already received chemotherapy regimens containing fluoropyrimidines, oxaliplatin, irinotecan, antiangiogenic agents, and, when indicated, epidermal growth factor receptor inhibitors. In addition, we examine those molecular pathways now included among the most promising areas of clinical research that will eventually drive innovative and more individualized treatment strategies.

**Abstract:**

Substantial improvements have characterized the systemic treatment of metastatic colorectal cancer (mCRC) over the past 20 years. Besides strong evidence that supports the use of RAS and BRAF status as prognostic and predictive indicators of disease and response, novel technologies have made possible the incorporation of emerging biomarkers for the management of mCRC. On one hand, the discovery of point mutations, amplifications, fusions, and gene expression profiles highlights the genomic and dynamic complexity of CRC. On the other, such discoveries are leading to newer biomarker-driven strategies that add to existing anti-epidermal growth factor receptor (EGFR) and anti-angiogenic approaches. In addition, the availability of a wide molecular profiling has relevant implications for patient prognosis and treatment benefits. Here, we will review the molecular underpinnings and clinical data supporting novel targeted treatments under development for refractory mCRC harboring BRAF mutations, KRAS G12C mutations, HER2 amplification, and less common molecular alterations, such as the re-arrangements of NTRK, ALK, and ROS1. Additionally, we will discuss novel strategies driving the rechallenge of EGFR antibodies and the incorporation of newer anti-angiogenic agents in the therapeutic armamentarium.

## 1. Introduction

Despite continuing improvements in cancer research, colorectal cancer (CRC) still ranks second among the leading causes of cancer-related deaths worldwide [1]. While surgery remains the primary treatment modality with a curative potential especially for early-stage disease, a large number of patients suffer from advanced/metastatic tumors, leading to a 5-year survival expectancy rate of less than 15% [2]. For these patients, systemic chemotherapy has long represented the mainstay of treatment, resulting in a median overall survival (OS) of 17–23 months [3,4,5]. Additional improvements in terms of OS have been driven by the combination of targeted therapies with standard chemotherapy regimens, such as antibodies against the epidermal growth factor receptor (EGFR) and the vascular endothelial growth factor (VEGF) [6,7,8].

In contrast to the old-fashioned, “one-size-fits-all” therapeutic approach, it is now clear that CRC is a highly heterogeneous disease, with several molecular subtypes and genetic alterations, often requiring selective treatment strategies and ultimately allowing (at least in a proportion of cases) for the implementation of a precision oncology care model. This contention is true now more than ever, with molecular biomarkers changing over time from simple, negative predictive/prognostic factors to useful therapeutic targets.

Herein we present the standard treatment options for refractory metastatic CRC (mCRC), and we discuss the latest research developments that may represent, pending data confirmation and/or regulatory approval, novel therapeutic options and innovative strategies in this setting. The immunological characterization of CRC and potential immunotherapeutic strategies have been reviewed elsewhere [9].

## 2. Current Systemic Treatments beyond Second-Line

Up to 30% of patients with mCRC are currently deemed eligible to receive three or more lines of therapy [10]. For patients who have already received combination chemotherapy regimens with 5-fluorouracil, oxaliplatin, irinotecan, VEGF-targeted agents, and, when indicated, EGFR-targeted agents, two further treatment options are available: regorafenib and trifluridine/tipiracil [11,12,13,14].

Regorafenib is an oral multi-kinase inhibitor (MKI) targeting multiple pathways, including angiogenesis (VEGF receptor [VEGFR] 1–3, TIE2), oncogenesis (KIT, RET, RAF-1, BRAF), and tumor microenvironment (platelet-derived growth factor receptors [PDGFR] and fibroblast growth factor receptors [FGFR]). Regorafenib was approved by the United States (U.S.) Food and Drug Administration (FDA) and the European Medicines Agency (EMA) in 2012 and 2013, respectively, following the positive results of the CORRECT trial [11]. This randomized, double-blind phase III study tested the efficacy of regorafenib at the dosage of 160 mg daily for 21 days in 28-day cycles, compared with a placebo in 760 patients with mCRC previously treated with all standard therapies. The study met its primary endpoint achieving a significant increase in OS and a small improvement in progression-free survival (PFS) with regorafenib [11]. The efficacy of regorafenib was confirmed in the CONCUR study, a randomized, placebo-controlled phase III trial recruiting 204 Asian patients with progressive mCRC who had received at least two previous treatments [12]. Unlike the CORRECT study, this trial also included patients who had not received prior biological treatment. The efficacy data of regorafenib are summarized in Table 1.

In both trials the occurrence of treatment-related adverse events (TRAEs) of any grade was observed in more than 90% of patients in the regorafenib arm. Roughly 50% of patients experienced grade 3 or higher TRAEs, among which hand-foot skin reaction, hypertension, fatigue, diarrhea, and laboratory abnormalities were the most frequent [11,12]. Moreover, dose reductions were required in more than 60% of patients treated with regorafenib, especially within the first 2 cycles. However, treatment discontinuation due to AEs was uncommon. Of note, no significant differences in quality of life (QoL) were seen between the two study arms in either trial.

A similar frequency and severity of AEs were reported in the CONSIGN study, a prospective, single-arm study recruiting a larger patient population (*N* = 2864) [15], and in the REBECCA trial (*N* = 654) [16], a multicenter study nested within a compassionate use program. These data led to the investigation of different regorafenib dosages, with the aim to improve the safety profile. The ReDOS study assessed a weekly dose escalation from 80 to 160 mg daily in the first cycle versus the standard dose, with the proportion of evaluable patients initiating the third cycle as the primary endpoint [17]. The study showed a significantly higher percentage of patients who started the third cycle at the dose of 160 mg daily in the experimental arm. Moreover, patients in the dose escalation arm experienced longer OS, better QoL, and fewer grade 3–4 AEs. In contrast, the phase II REARRANGE trial failed to demonstrate that either a reduced dose (120 mg daily) or an intermittent schedule (160 mg daily, 1 week on, 1 week off) during the first cycle of regorafenib could reduce the risk of grade 3–4 AEs [18].

Currently, no validated predictive biomarkers are available for patients treated with regorafenib. Findings from the analysis of circulating DNA and protein biomarkers of the CORRECT trial showed that KRAS and PIK3CA mutation status did not have any predictive value. Although high levels of the sTIE1 plasma protein seemed to predict a greater regorafenib benefit, the association was not significant in the multivariate analysis [19]. Of note, according to a separate post-hoc analysis of the same study, the free triiodothyronine (FT3)/free thyroxine (FT4) ratio may be a useful surrogate prognostic factor [20].

Trifluridine/tipiracil is an oral fluoropyrimidine consisting of two compounds, trifluridine, a cytotoxic nucleic acid analogue, and tipiracil, a thymidine phosphorylase inhibitor that blocks the trifluridine enzymatic degradation. Trifluridine/tipiracil was approved by the FDA and the EMA in 2015 and 2016, respectively, following the positive results of the RECOURSE trial, a multicenter randomized phase III study (*N* = 800) comparing trifluridine/tipiracil to a placebo in previously treated mCRC patients [13]. Of note, prior regorafenib use was reported in 17% and 20% of patients in the trifluridine/tipiracil and in the placebo arm, respectively. In the investigational arm, patients received trifluridine/tipiracil at the dosage of 35 mg/m^2^ twice daily on a 28-day schedule (5 days on and 2 days off for each of the first 2 weeks followed by a 2-week rest period). The study met its primary endpoint reaching a greater benefit in median OS and slightly longer median PFS in the trifluridine/tipiracil arm compared to the placebo arm. Overall, grade ≥ 3 AEs occurred more frequently in the trifluridine/tipiracil group (69%) compared to the placebo group (52%). Among patients receiving trifluridine/tipiracil, neutropenia was the most common AE (grade ≥ 3 in 38% of patients), although only 4% experienced febrile neutropenia. In the experimental arm, TRAEs required dose modifications in 14% of cases, and treatment discontinuation in 4%. A similar phase III study, the TERRA trial, was conducted in Asia [14]. The study enrolled 406 patients previously treated with ≥2 lines of therapy. In contrast to the RECOURSE trial, this study allowed the enrolment of patients untreated with prior biological agents (anti-VEGF or anti-EGFR therapy). This study showed greater benefit from trifluridine/tipiracil compared to the placebo, with efficacy and safety data similar to those observed in the RECOURSE trial. The efficacy data of trifluridine/tipiracil are summarized in Table 1.

Interestingly, post-hoc analyses showed the association between a decreased neutrophil count and trifluridine/tipiracil efficacy, suggesting the role of neutropenia as a potential predictive factor [21,22]. Moreover, a further post-hoc exploratory analysis showed an increased OS and PFS with trifluridine/tipiracil for a subgroup of patients with good prognostic characteristics defined by low tumor burden (<3 metastatic sites), less aggressive/indolent disease (≥18 months from diagnosis of first metastasis to randomization), adequate organ function, and Eastern Cooperative Oncology Group (ECOG) performance status (PS) 0–1 [23].

A systematic review and network meta-analysis showed no statistically significant difference between regorafenib and trifluridine/tipiracil in terms of OS and PFS, with a better safety profile for trifluridine/tipiracil [24]. Another meta-analysis achieved similar results, further showing that the regorafenib dose escalation schedule (as investigated in the ReDOS trial) was superior to best supportive care, and led to numerically longer OS compared with regorafenib 160 mg or trifluridine/tipiracil [25].

Despite an increase of OS provided by regorafenib and trifluridine/tipiracil, most mCRC patients will eventually experience primary or secondary resistances to all standard treatments. These observations raise the possibility to increase treatment efficacy through a different approach based on patient stratification derived from tumor biology.

## 3. Recent Developments and Ongoing Clinical Trials

### 3.1. Targeting EGFR (Rechallenge)

Anti-EGFR monoclonal antibodies, namely cetuximab and panitumumab, represent standard targeted therapies for patients with RAS wild-type mCRC. While they are generally used in combination with first-line chemotherapy, there is growing evidence suggesting their potential utility when given in the refractory setting as part of rechallenge treatment strategies.

Rechallenge is defined as the reintroduction of the same therapy to which tumors were initially sensitive (i.e., objective response or stable disease [SD]), before showing resistance (progressive disease [PD]) while on treatment [26]. Extended-RAS alterations represent the main anti-EGFR acquired resistance mechanism. Resistance could be potentially reverted by the exposure to other therapeutic agents due to the decay of altered RAS clones upon anti-EGFR treatment withdrawal. In particular, the length of intervening treatment plays a pivotal role to the re-establishing of anti-EGFR sensitivity [27]. Secondary resistance could also be associated with the emergence of activating mutations in EGFR downstream effectors and mutations in the EGFR extracellular domain [28]. The development of the S492R mutation in the EGFR binding epitope has potential implications for treatment since it selectively disrupts cetuximab, but not panitumumab binding, and supports rechallenge with panitumumab in patients previously treated with cetuximab and developing an S492R mutation [29].

In a prospective phase II trial, Santini et al. enrolled 39 patients with KRAS wild-type tumors and confirmed a partial response (PR) or SD for at least 6 months during first-line therapy with cetuximab plus irinotecan-based chemotherapy, followed by disease progression. Patients were retreated beyond second- and third-line with irinotecan-based chemotherapy and cetuximab. The overall response rate (ORR) was 53.8%, including PR in 48.7% of patients and complete response (CR) in 5.1%. The median PFS was 6.6 months (95% CI, 4.1–9.1%) [30].

The efficacy of the irinotecan plus cetuximab rechallenge was also evaluated in the prospective multicenter phase II JACCRO CC-08 trial. In 34 enrolled patients, the 3-month PFS rate, the primary endpoint of the study, was 44.1% (95% CI, 27.4–60.8%). One patient achieved PR and 18 patients SD with a disease control rate (DCR) of 55.9% (95% CI, 7.9–72.8%). A post-hoc subgroup analysis showed an association between the duration of the cetuximab-free interval (CFI) and rechallenge benefit, with the median PFS being 4.6 months and 2.1 months (HR 0.40; 95% CI, 0.18–0.86; *p* = 0.020), in the long and short CFI group, respectively [31]. However, this association was not confirmed, and inconsistent data were reported by a multi-institutional retrospective real-world study [32].

In 2018, the preliminary data of the E-Rechallenge study were presented. In 33 patients rechallenged with cetuximab and oxaliplatin, the PR was 15.6% (95% CI, 5.3–32.7%), SD 40.6% (95% CI, 23.6–57.6%), and PD 43.8% (95% CI, 26.4–62.3%). The median PFS was 88 days (range 62–113 days) and the median OS 262 days (range 195–307 days) [33].

The efficacy of the cetuximab rechallenge in combination with tivantinib, a MET inhibitor, was prospectively evaluated in a phase II trial. This study evaluated 41 patients with MET-high, KRAS wild-type mCRC, who were treated with ≥1 prior systemic therapy, with at least SD on the last treatment regimen containing cetuximab or panitumumab and tumor progression within 3 months before enrollment. However, the study did not meet its primary endpoint of ORR [34].

The phase II CRICKET trial tested the cetuximab rechallenge in 28 irinotecan-pretreated, RAS/BRAF wild-type mCRC patients who had received an anti-EGFR-based first-line therapy, with at least PR for > 6 months, followed by PD [35]. After progression to the second-line therapy, patients were retreated with an irinotecan-based chemotherapy and cetuximab. The overall response rate was 21% (95% CI, 10–40%) with a median OS of 9.8 months (95% CI, 5.2–13.1 months) and a median PFS of 3.4 months (95% CI, 1.9–3.8 months). Liquid biopsy samples were collected at the time of the rechallenge for retrospective analyses. Notably, all PRs were observed in patients with RAS wild-type circulating tumor DNA (ctDNA). The median PFS was 4 months and 1.9 months (hazard ratio [HR] 0.44; 95% CI, 0.18–0.98; *p* = 0.03) in patients with RAS wild-type and RAS mutant ctDNA, respectively. These data suggest that liquid biopsy/ctDNA analysis may be a useful tool to identify those patients who are most likely to benefit from a rechallenge strategy.

A further step in this direction is represented by the ongoing phase II CHRONOS study. The purpose of this study is to assess the efficacy of rechallenge with panitumumab in extended-RAS wild-type mCRC patients with ctDNA-confirmed secondary resistance to anti-EGFR treatment. Preliminary data were presented at the ASCO Annual Meeting 2021. Fifty-two patients were screened by liquid biopsy and 36 (69%) had ctDNA negative for RAS/BRAF/EGFR mutations. The primary endpoint was met with an ORR of 30% (95% CI, 12–47%), with 8/27 and 11/27 PR and SD, respectively. The median PFS was 16 weeks [28].

Other studies are investigating the combination of cetuximab and the anti-protein programmed death-ligand 1 (PD-L1) antibody avelumab as a novel rechallenge strategy. When combined with immune checkpoint inhibitors (ICI), cetuximab may activate a functional cross-talk between natural killer and dendritic cells, recruit cytotoxic T cells in the tumor microenvironment and prime the immune system to be more sensitive to ICI treatment. A combined therapy with cetuximab plus avelumab may be able to activate both innate and adaptive immune responses.

The final results of the phase II CAVE (cetuximab rechallenge plus avelumab) trial were presented at the ASCO Annual Meeting 2021. In 77 enrolled patients, the median OS was 11.6 months (95% CI, 8.4–14.8) and the median PFS was 3.6 months (95% CI, 3.2–4.1) with a manageable safety profile [36].

Further phase II trials are ongoing to evaluate different rechallenge strategies (Table 2). Most of these studies compare rechallenge treatment with standard of care and their results will be potentially relevant to designing personalized treatment algorithms and sequence strategies.

In conclusion, based on the above-mentioned data, the anti-EGFR rechallenge seems a promising strategy with a good safety profile, and liquid biopsy may have a role as a tool for patient selection. However, these preliminary results should be confirmed in prospective randomized trials.

### 3.2. Targeting BRAF

BRAF mutations are detected in 8–10% of mCRC patients. Of these, >90% are missense mutations occurring in codon 600 and determining an aminoacidic substitution of a valine for a glutamic acid (V600E). In mCRC, BRAF V600E mutations represents a poor prognostic factor, leading to a median OS between 10 and 20 months [37]. A relatively higher incidence of BRAF V600E mutations in older ages may contribute to a worse OS for patients carrying this mutation [38].

Of note, high microsatellite instability (MSI-H) and BRAF V600E mutations often overlap and up to 50% of BRAF V600E mutant CRCs have an MSI-H status [39]. Colorectal cancers harboring such features are always sporadic and characterized by somatic hypermethylation across the tumor cell genome, including gene silencing by methylation of the MLH1 promoter. In contrast, MSI status in the absence of a BRAF V600E mutation may indicate a Lynch syndrome-associated CRC.

In pretreated mCRC with BRAF V600E mutations and concomitant MSI, ICIs portend ORR that are durable and numerically superior to those expected with standard therapies [40,41]. However, the largest proportion of BRAF mutant patients being treated with ICI was roughly one fourth of the entire DNA Mismatch Repair–Deficient/MSI–H cohort in the CheckMate 142 trial, thereby precluding any definitive conclusion for this subset of patients [41].

Unfortunately, the encouraging results with single-agent BRAF inhibitors in melanoma, thyroid cancer, and lung cancer were not confirmed in previously treated BRAF V600E-mutant CRC. The low response rates achieved by either monotherapy with BRAF or EGFR inhibitors have led to further studies investigating multi-target treatment approaches. Among various anti-BRAF and anti-EGFR combinations explored, the BRAF inhibitor encorafenib combined with cetuximab showed promising activity in early clinical trials [42]. Furthermore, adding an MEK inhibitor to BRAF inhibition has also been found to increase the inhibition of the MAPK pathway and produce potentially greater antitumor activity in preclinical and early clinical studies [43].

Consequently, additional efforts were undertaken to develop the combination of BRAF inhibitors, MEK inhibitors, and EGFR inhibitors.

In the phase III BEACON trial, the addition of encorafenib and binimetinib (a MEK inhibitor) to cetuximab significantly improved the median OS (9.0 months; 95% CI, 8.0–11.4), as compared to cetuximab plus chemotherapy (5.4 months; 95% CI, 4.8–6.6), translating into a significantly lower risk of death (HR 0.52; 95% CI, 0.39–0.70; *p* < 0.001) [44]. Updated results for OS, PFS and ORR after 6 additional months of follow-up were consistent [45]. Given the small proportion of patients with BRAF mutant MSI-H CRC (10%), the benefit deriving from the novel regimen has still to be established in this subgroup of patients.

In contrast to V600E BRAF mutations, non-V600E mutations in CRC patients are less frequent and they are found in nearly 2% of patients [46]. In all, more than 200 non-V600E BRAF mutations (also known as atypical BRAF mutations) have been discovered.

Non-V600E mutations tend to cluster within well differentiated tumors, with a preferential location on the left side. No significant associations with MSI status, nor with tumor stage, have been reported and they seem to be associated with a better prognosis compared to V600E BRAF mutated CRC [47].

Nevertheless, the level of kinase activity and mechanisms of activation further dissect non-V600E BRAF mutations into distinct classes (Class 2 and Class 3) with prognostic and therapeutic implications. While Class 2 mutations confer an intermediate kinase activity linked to poor prognosis, Class 3 mutations are RAS dependent and exhibit low or absent kinase activity. Of note, sensitivity to EGFR inhibitors has been shown in preclinical models exhibiting Class 3 mutations [48].

### 3.3. Targeting KRAS

Approximately 45% of CRCs have KRAS mutations which are associated with a lack of response to EGFR targeting therapies and poor survival outcomes [49]. Decades of remarkable efforts to inhibit RAS activation first led to farnesyltransferase inhibitors, which were intended to prevent RAS membrane localization and subsequent activation. Nevertheless, this drug class showed no benefit in OS versus standard supportive therapy [50]. Later investigations mostly explored RAS downstream targets belonging to the MAPK cascade (MEK, ERK, pan-RAF, SHP2, etc.) and/or PI3K cascades [51], but these agents tested as monotherapy carried disappointing results. Given these limitations, parallel efforts focusing on direct RAS inhibition were pursued in order to provide a more comprehensive inhibition of the downstream activity. A major hurdle was indeed the high affinity of RAS for GTP/GDP and the absence of known allosteric sites suitable for small-molecule targeting.

The KRAS G12C mutation is reported in 3% of CRCs and associated with worse survival outcomes, though data are conflicting [52]. In 2013, novel insights into the structure and biochemical properties of mutant KRAS G12C have subsequently led to the discovery of covalent inhibitors, including AMG510 and MRTX849, being currently tested in CRC.

Interestingly, these compounds have no activity against other KRAS mutations, such as G12V and G12D, which are more common in CRC. In the first in-human phase I study, AMG510 was evaluated in a dose-escalation design in patients with refractory KRAS G12C mutated solid tumors (NCT03600883). In the CRC cohort, a confirmed PR was detected in 3 of 42 patients (7.1%) while the DCR was 73.8%. The median OS had not been reached after a median follow-up of almost 8 months and the median PFS was 4 months (range 0.0–11.1) [53]. On the other hand, in the recently presented KRYSTAL-1 study (NCT03785249), a multicenter phase I/II study of MRTX849 in patients with advanced solid tumors that harbor a KRAS G12C mutation, 3 out of 18 evaluable patients had a confirmed PR, with a DCR observed in 94% of CRC patients [54].

In line with the previously observed differential response to BRAF inhibitors of BRAF V600E mutated melanoma and mCRC, the activity of AMG510 in KRAS G12C mutated CRC is substantially lower than in non-small cell lung cancer (NSCLC). In fact, in CRC cell lines, KRAS G12C inhibition was shown to induce greater EGFR-mediated, MAPK pathway reactivation, thus supporting EGFR as a valuable target to overcoming resistance to KRAS covalent inhibitors in mCRC.

This hypothesis has been preclinically validated using the combination of cetuximab and AMG510. Significant tumor reduction compared to either agent alone was detected, including a 73-day sustained response in one of the models. These data support the rationale for the full vertical inhibition of EGFR-KRAS G12C for optimal efficacy, as already observed with other MAPK activating alterations in CRC [55]. Accordingly, a trial of MRTX849 in combination with cetuximab is ongoing (NCT03785249). In addition, a randomized phase III study of second-line MRTX849 in combination with cetuximab versus chemotherapy (FOLFOX or FOLFIRI) in mCRC with the KRAS G12C mutation therapy is currently recruiting (NCT04793958).

### 3.4. Targeting HER2

The human epidermal growth factor receptor 2 (HER2) belongs to the EGFR family and is ordinarily activated by binding to specific ligands and dimerization with other EGF family receptors [56]. The overexpression of HER2, commonly caused by the amplification of the ERBB2 gene, allows for the activation of replication signals even in the absence of ligand-bound dimerization partners [57]. In mCRC, HER2 overexpression varies substantially in published series [58], with RAS wild-type and rectal cancer tumors showing the highest prevalence (approximately 5–8%).

Two landmark studies paved the way for the development of HER2 as a therapeutic target in mCRC. The HERACLES study was a single-arm, open-label phase II trial in which 27 treatment-refractory patients had received dual HER2 inhibition with trastuzumab and lapatinib [59]. The eligibility criteria included HER2 overexpression or amplification (defined as IHC 3+ score in > 50% of cells or 2+ score and FISH HER2:CEP17 ratio > 2.0 in > 50% of cells), and prior exposure to anti-EGFR therapies. The study demonstrated a 30% ORR, meeting the pre-defined positivity threshold, with one patient maintaining a CR for 7 years [60]. The median survival outcomes (PFS 4.7 months, OS 10.0 months) were also encouraging especially for a population that had received a median number of 5 prior to the lines of therapy. Of note, a retrospective analysis showed a strong correlation between HER2 amplification in tissue and in plasma (96.6%), and identified a ctDNA-based, copy number threshold to predict anti-HER2 treatment benefits [61].

MyPathway was a non-randomized, open-label basket trial including a cohort of 57 patients with HER2-amplified mCRC who were treated with a combination of pertuzumab and trastuzumab [62]. Patients in this study had to have received at least one prior treatment and were not selected based on RAS status. The median number of previous therapies was four and 23% of patients had KRAS mutant tumors. Consistently, with the activity shown in the HERACLES study, dual anti-HER2 blockade led to a 32% ORR, with four patients having durable responses lasting more than 1 year. Interestingly, one PR was observed in a KRAS mutant patient. The median PFS was 2.9 months and the median OS was 11.5 months. The activity of the pertuzumab-trastuzumab combination was confirmed in a small HER2-amplified, RAS wild-type, anti-EGFR pre-treated population from the Japanese TRIUMPH study [63]. Notably, this study was the first to include patients based on a ctDNA analysis, ORR being 35% and 33% in the tissue-positive and ctDNA-positive population, respectively.

Based on the encouraging results from these studies, the interest for the clinical potential of the therapeutic blockade of HER2 in this setting has rapidly increased and other compounds have recently been developed.

Tucatinib, a novel anti-HER2 oral treatment, has shown promising activity in combination with trastuzumab in the non-randomized part of the phase II, open-label MOUNTAINEER trial [64]. Patients had HER2 amplification/overexpression and had to have previously been treated with standard chemotherapy and anti-VEGF agents. The combination was well tolerated, and among 22 evaluable patients, the ORR was 55%, with the median PFS of 6.2 months and the median OS of 17.3 months. The trial is currently enrolling in the randomized part of the study, testing tucatinib plus trastuzumab versus tucatinib monotherapy [65].

Further promising results have recently been published regarding trastuzumab deruxtecan, an antibody drug conjugate including a humanized anti-HER2 antibody linked to a novel topoisomerase inhibitor [66]. The DESTINY-CRC01 trial enrolled 78 RAS/BRAF wild-type mCRC patients refractory to standard treatments. Previous anti-HER2 therapy was not an exclusion criterion [67,68]. Patients were divided into three cohorts based on the degree of HER2 overexpression/amplification: cohort A (*N* = 53) immunohistochemistry (IHC)3+/IHC2+ and in situ hybridization (ISH) positive; cohort B (*N* = 15) IHC2+ and ISH negative; cohort C (*N* = 18) IHC1+. In cohort A, the ORR was 45.3% (with a median PFS of 6.9 months), while no patient showed an objective response in the other two cohorts. Of note, the drug was also active in patients previously treated with anti-HER2 agents, with a median PFS of 4.3 months. Eight patients (9.3%) in the study population experienced interstitial lung disease, and in three cases this had a fatal outcome. The DESTINY-CRC02 trial, a phase II study testing 2 different doses of trastuzumab deruxtecan, has recently started enrolment [69].

Several other HER2-targeting agents, such as margetuximab, neratinib, pyrotinib, and poziotinib are currently in earlier phases of development, and more data about their potential in mCRC are eagerly expected.

### 3.5. Targeting NTRK, ALK and ROS1 Fusion

Neurotrophin receptor tyrosine kinase proto-oncogenes NTRK1, NTRK2 and NTRK3 encode for TRKA, TRKB and TRKC, respectively, which are members of a family of transmembrane receptors activated by different ligands: neurotrophin nerve growth factor (NGF) for TRKA, neurotrophin 4 (NT-4) and brain-derived neurotrophic factor (BDNF) for TRKB, and neurotrophin 3 (NT-3) for TRKC [70]. In physiological conditions, they exert their biological functions through homodimerization and activation of downstream signaling pathways (mainly RAS-MAPK and PI3K-AKT), ultimately regulating cell survival and proliferation of neural precursors during embryogenesis but also synaptic strength and plasticity in adult neuronal cells [71]. Several alterations of the NTRK genes have been described in human cancers, such as mutations, amplifications, and splice variants, but fusions are the most relevant and common oncogenic events, with the NTRK kinase domain being constitutively activated as a result of inter- or intra-chromosomal rearrangements [72,73].

Larotrectinib and entrectinib have recently been approved as tissue-agnostic drugs in NTRK-rearranged tumors by the EMA and FDA [74]. Larotrectinib is a highly selective oral TRK inhibitor which was tested in a phase I/II trial of 55 pediatric and adult cancer patients harboring NTRK fusions, showing an impressive ORR of 75% and good tolerability [75]. Entrectinib, an oral pan-TRK, ROS1 and ALK kinase inhibitor, was tested in 54 adult, NTRK rearranged cancer patients, yielding an ORR of 57% and showing a safety profile comparable with larotrectinib [76].

In CRC, NTRK fusions are rare, occurring in less than 2.5% of metastatic patients, mainly with right-sided, MSI-H, and RAS/BRAF wild-type tumors [77,78]. Although limited by the small numbers, studies suggest an association between NTRK fusions, reduced benefit from anti-EGFR therapies, and worse outcome [77]. In the studies by Drilon et al. and Doebele et al., one out of four mCRC patients who were enrolled in each trial had a PR with larotrectinib and entrectinib, respectively [75,76]. In a recent update of the phase II NAVIGATE trial reporting on the outcome of ten mCRC patients (seven of whom had MSI-H tumors), larotrectinib treatment was associated with an ORR of 50%, DCR of 100%, median duration of response of 15.5 months, median PFS of 5.5 months and median OS of 29.4 months [79]. While prolonged, the benefit from currently approved NTRK inhibitors is eventually impaired by the occurrence of tumor resistance, which can involve on-target kinase domain mutations or off-target mechanisms [80,81]. Next-generation NTRK inhibitors, such as repotrectinib or selitrectinib, are currently under investigation to revert acquired resistance to entrectinib or larotrectinib [76].

The anaplastic lymphoma kinase (ALK) and ROS1 genes encode for the homonym tyrosine kinase receptors. Following activation by their respective ligands (FAM150A and FAM150B for ALK, neural epidermal growth factor-like like 2 for ROS1), they regulate a number of cellular functions through different signaling pathways [82,83,84]. While rare, alterations of these genes, including mutations, fusions, and amplifications have been described in several tumor types [85,86]. As previously described for NTRK, fusions of ALK (generally with the EML4 partner) and ROS1 lead to the constitutive, oncogenic activation of the kinase domain, thereby representing an interesting therapeutic target. In line with this rationale, ALK and ROS1 inhibitors have dramatically improved survival and QoL of patients with NSCLC harboring these alterations [87,88].

In mCRC, ALK and ROS1 fusions are extremely rare, with an overall prevalence of ≤1% (8, 20) [77,89]. As for NTRK, ALK and ROS1 fusions have been found to be associated with microsatellite instability and poor prognosis [77,90]. While data from prospective clinical trials are lacking, case reports support the contention that, especially when second and third generation inhibitors (such as ceritinib, alectinib, and lorlatinib) are used, ALK inhibition may be a valid therapeutic option for ALK translocated mCRC patients, with the potential to induce durable responses [91,92,93].

### 3.6. Targeting Angiogenesis

Angiogenesis is the complex mechanism that ultimately allows localized tumors to grow through the formation of new vessels, which supply oxygen and nutrients to cancer cells [94]. VEGF is certainly one of the most important molecules involved in tumor angiogenesis and it became the first target for the anti-angiogenic drug development [95]: to date, bevacizumab, ramucirumab, and aflibercept, which are respectively a humanized antibody against VEGF-A, a humanized antibody against VEGFR2 and a decoy receptor binding VEGF-A, are approved for use in mCRC patients. However, several other receptors and pathways are involved in tumor angiogenesis: FGFRs, PDGFRs, transforming growth factor-beta receptor (TGFβR), and hepatocyte growth factor receptor (HGFR, most known as MET), just to name a few [96]. Regorafenib is the precursor in its field [97]; however, the limited benefit shown in the phase III clinical trials [11,12] have pointed out the need of new drugs able to overcome and prevent primary and secondary resistance, respectively, in mCRC.

New molecules targeting angiogenesis have recently been tested in (chemo)refractory mCRC (Table 3). Among these, fruquintinib is the one with the most advanced data, having received regulatory approval in China and been granted fast track status by the FDA. This oral VEGFR1–3 selective inhibitor [98] was compared against a placebo in a randomized phase III trial (FRESCO, *N* = 416) conducted in China. Eligibility was restricted to patients who had received at least two prior lines of chemotherapy with or without bevacizumab or aflibercept, whereas prior use of VEGFR inhibitors was not allowed [99]. The primary endpoint of the study was met, with the median OS being statistically significantly longer in the investigational than in the placebo arm (9.3 vs. 6.6 months; HR 0.65; *p* < 0.001). Moreover, patients treated with fruquintinib had better median PFS (3.7 vs. 1.8 months; HR 0.26; *p* < 0.001), ORR (4.7% vs. 0%; *p* = 0.01), and DCR (62.2% vs. 12.3%; *p* < 0.001). Of note, while the proportion of patients receiving prior antiangiogenic therapies was relatively low (approximately 30%), subgroup analyses did not show any interaction between treatment efficacy and prior exposure to either bevacizumab or aflibercept. The safety profile of fruquintinib was in line overall with the expectations, with higher rates of grade ≥3 AEs (61.2% vs. 19.7%) and serious AEs (15.5% vs. 5.8%) being reported among patients in the investigational arm. Most common grade ≥3 AEs included hypertension (21.2%), hand-foot skin reactions (10.8%), and proteinuria (3.2%). One of the main questions remaining about the use of fruquintinib in mCRC is whether these results can be reproduced in Western populations. In this regard, a multicenter randomized phase III trial (FRESCO-2) is currently ongoing in Europe, the U.S., and Japan to assess the efficacy and safety of this agent versus the placebo in the refractory setting. Notably, and in contrast with the FRESCO trial, eligible patients must have progressed on, or be intolerant to, all available agents including either trifluridine/tipiracil or regorafenib (NCT04322539) [100].

Anlotinib is an oral multi-kinase inhibitor with a number of targets including, among others, VEGFR1-3, PDGFRα-β and FGFR1-3 kinases [106]. Also, preclinical data showed that, in addition to antiangiogenic effects, this drug can exert antitumor activity by inhibiting the AKT/ERK signaling pathway [107]. In the recent phase III ALTER0703 trial from China, anlotinib was compared against placebo in chemorefractory mCRC. While the investigational treatment was associated with better ORR, DCR, and PFS, the study failed to meet the primary endpoint, showing no OS benefit (HR 1.02; *p* = 0.87). Notably, and possibly warranting further investigation, a statistically significant improvement in OS was observed in the subgroup of patients with RAS/BRAF wild-type tumors. Main grade ≥3 AEs included hypertension (21%), increased γ-GT (7%) and hand-foot syndrome (6%) [101]. An ongoing phase II trial is evaluating anlotinib combined with oxaliplatin-based chemotherapy in the first-line setting [108].

Apatinib is a highly selective oral inhibitor of VEGFR2 [109], with a putative activity as a multidrug resistance reverser as well [110]. A single-arm phase II trial of this compound in Chinese chemorefractory mCRC patients reported a DCR of 69% and a median OS of 9.1 months, with hypertension (13%), hand-foot syndrome (10%), and thrombocytopenia (10%) being the most frequent grade ≥3 AEs. The lack of a control arm and the high proportion of anti-angiogenic-naïve patients make the interpretation of these data challenging [102]. An ongoing phase II study is investigating the efficacy of apatinib in combination with S-1 in the same setting (NCT03397199).

Cabozantinib is an oral MKI with a wide range of actions, since it suppresses many tyrosine kinases, such as VEGFR2, MET, RET, KIT, AXL, and FLT3 [111]. In a single-arm phase II trial from the U.S., cabozantinib showed encouraging efficacy, with a 12-week PFS rate of 34% (as compared with the historical 13% for regorafenib in the CORRECT trial) and a DCR at 6 weeks of 73% in heavily pretreated patients. The safety profile was similar overall to that observed with the same agent in other tumor types, and up to 41% of patients were reported to experience one or more serious AEs [103]. Based on the notion that MET amplification is one of the established drivers of anti-EGFR resistance, the combination of cabozantinib and panitumumab was recently tested in a phase Ib trial including anti-EGFR naïve patients with refractory, RAS wild-type tumors. While the treatment safety profile was acceptable, the activity data (i.e., ORR 16%) do not appear different from those achievable with a single agent panitumumab in a similar patient population [112].

Similar to cabozantinib, nintedanib is an oral compound targeting multiple kinases, such as VEGFR1-3, PDGFRα-β, FGFR1-3, RET, and FLT3 [113]. Despite preliminary signals of anti-tumor activity in early-phase studies, this agent failed to become a potentially useful asset for the management of advanced CRC. The randomized, phase III LUME-Colon 1 trial (*N* = 768), compared nintedanib with a placebo in chemorefractory mCRC patients (37% and 14% of whom had been previously exposed to regorafenib and trifluridine/tipiracil, respectively). PFS and OS were co-primary endpoints. While a statistically significant (although marginal) PFS advantage in favor of the investigational treatment was reported (1.5 vs. 1.4 months; HR 0.58; *p* < 0.0001), no difference in OS was observed (6.4 vs. 6.0 months; HR 1.01; *p* = 0.87) [104]. The risk of grade ≥3 AEs increased from 28.6% to 41.9% with nintedanib, the most common being liver-related toxicities. Smaller randomized trials were also conducted to test this anti-angiogenic agent in combination with standard chemotherapy, but interpretation of the results is difficult due to the premature discontinuation of the same and lack of statistical power [114].

Famitinib is an oral inhibitor of VEGFR2, PDGFRβ, and KIT receptors [115]. In a randomized, phase II trial from China (*N* = 154), the administration of this compound was associated with an improvement in PFS (2.8 vs. 1.5 months; HR 0.60; *p* = 0.004) and DCR (59.8% vs. 31.4%; *p* = 0.002) as compared with a placebo in a population of chemorefractory mCRC patients [105]. Grade ≥ 3 AEs occurred in 51.5% and 36.4% of patients, respectively. Interestingly, thrombocytopenia (10.1%), and neutropenia (9.1%) were among the most common toxicities.

In addition to the development of new agents, novel combinations, such as the ones including bevacizumab and trifluridine/tipiracil, are currently being evaluated in mCRC, based on the results of preclinical and early-phase clinical studies showing promising antitumor activity and a manageable safety profile [116,117,118]. A recent randomized phase II trial tested trifluridine/tipiracil plus bevacizumab versus trifluridine/tipiracil alone in 93 chemorefractory patients [119]. Combination treatment was superior in terms of the primary endpoint PFS (4.6 vs. 2.6 months; HR 0.45; *p* = 0.001) and OS (9.4 vs. 6.7 months; HR 0.55; *p* = 0.028). The same combination was investigated in parallel with capecitabine plus bevacizumab in the randomized, non-comparative, phase II TASCO1 trial [120]. This study enrolled patients who had not received prior palliative treatment and who were considered ineligible for full-dose combination chemotherapy. The median PFS, median OS, and ORR were 9.2 months, 18 months, and 34%, respectively. Based on these encouraging results, two phase III trials have recently been launched: SOLSTICE (trifluridine/tipiracil plus bevacizumab versus capecitabine plus bevacizumab in previously untreated mCRC patients who are not candidates for intensive therapy, NCT03869892) and SUNLIGHT (trifluridine/tipiracil plus bevacizumab versus trifluridine/tipiracil monotherapy in refractory mCRC patients) [121].

In mCRC, mounting evidence is broadening the scope of established biomarkers, which currently provide a rationale framework for improved outcomes obtained by targeting the marker itself. Although the majority of such biomarkers has a low prevalence, along with an expanding therapeutic armamentarium, the list is overall increasing. From this standpoint, mCRC will eventually be regarded as an umbrella diagnosis recapitulating less frequent disease subtypes with specific targeted treatment options.

## 4. Discussion

For many years, CRC has lagged behind many other tumor types with regard to the discovery of oncogenic drivers and molecular targets with therapeutic relevance. Recently, however, some progress has been made, and the molecular taxonomy of this disease has enriched for small but clinically relevant subgroups. As outlined in this review, and in addition to mismatch repair deficiency/microsatellite instability and tumor mutational burden (the discussion of which was beyond the scope of the manuscript), mutations of KRAS and BRAF, overexpression/amplification of HER2, and fusions of NTRK, ALK and ROS1 provide an opportunity for novel therapeutic approaches with interesting, and in some cases unprecedented, results. Furthermore, new compounds and repurposing/combination strategies have renewed the interest for long-established targets, such as EGFR and angiogenesis. As is often the case when new evidence emerges, many challenges remain to translate data from clinical trials into direct applications for clinical practice.

Confirmation of preliminary results in larger studies is needed. Most of the data here presented are from basket trials or small, single-arm phase II studies, and the interpretation of the role of new therapies within the context of the pre-existing evidence is difficult. One of the key questions is whether exceptional and historically unparalleled findings from a small, uncontrolled prospective series should be considered as ranking high enough in the evidence hierarchy to challenge established approaches, which are based on large, randomized phase III trials. It is not uncommon to see highly promising results from early investigations (where the risk of selection bias is high and appropriate controls are lacking), not being reproduced in later studies. It is clear, though, that the rare frequency of some therapeutic targets precludes running larger conventional studies, and the pursuit of an efficient precision oncology research model largely depends on the implementation and endorsement by clinical researchers and regulatory agencies of unconventional models of drug development. Of course, striking a good balance between the need to accelerate the drug approval process and the obligation to respect some basic principles of cost-effectiveness is paramount to guaranteeing the credibility and sustainability of the entire system.

Another key challenge is to understand how to best position some of these new agents into the therapeutic algorithm of mCRC. For obvious reasons, most of the available data at this stage are from the refractory setting. It is established, however, that only a minority of patients are suitable for third or subsequent lines of therapy, such a substantial treatment dropout precluding in most cases the chance of receiving highly-active compounds that may eventually perform better than standard therapy. This is especially true if the therapeutic target is also an unfavorable prognostic biomarker, which is for instance the case of BRAF. Theoretically, an earlier use of novel, molecularly-targeted therapies should have even a stronger prognostic impact both at the individual and population level, but evidence should be rapidly generated to confirm this assumption (the overall performance of standard-of-care first- and second-line options being by far superior than that of currently available third- or subsequent line therapies), to provide guidance regarding the optimal sequencing of available therapies, and ultimately inform physicians in the treatment selection process.

Finally, at least for those compounds that target tumor molecular aberrations, routine implementation and standardization of molecular testing procedures are key to ensuring an efficient model of patient stratification and treatment allocation. It is clear, however, that a mismatch still exists between the pace of drug testing and the advancement of companion diagnostics. Long-established, clinically relevant biomarkers are not yet routinely (or promptly) tested in some centers, and no universal consensus or guidelines exist for the analysis and interpretation of novel biomarkers, such as ctDNA, which remain largely investigational. Furthermore, more studies are needed to refine patient selection, identify resistance mechanisms, and test alternative upfront strategies or molecularly-informed sequential approaches to overcome these.

## 5. Conclusions

Regorafenib and trifluridine/tipiracil represent standard treatment options for chemotherapy-refractory mCRC, but their efficacy is limited, and the prognosis of these patients remains poor. A number of novel, mostly molecularly-driven compounds have recently emerged, with the potential to substantially improve outcomes and set new standards of care in this setting. Many challenges still exist to seeing tangible signs of such advances. The hope is that we are up to the task and able to keep the momentum going to allow the rapid implementation of novel treatments, and to eventually target the substantial proportion of patients who cannot benefit yet from personalized management strategies.

## Figures and Tables

**Table 1:** Key phase III efficacy data of regorafenib and trifluridine/tipiracil.

	CORRECT		CONCUR		RECOURSE		TERRA	
	R	P	R	P	T/T	P	T/T	P
Patients (*N*)	505	255	136	68	534	266	271	135
mOS (months)	6.4	5.0	8.8	6.3	7.1	5.3	7.8	7.1
HR (95% CI); *p*	0.77 (0.64–0.94); 0.0052		0.55 (0.4–0.77); 0.00016		0.68 (0.58–0.81); <0.001		0.79 (0.62–0.99); 0.035	
mPFS (months)	1.9	1.7	3.2	1.7	2.0	1.7	2.0	1.8
HR (95% CI); *p*	0.49 (0.42–0.58); 0.0001		0.31 (0.22–0.44); <0.0001 *		0.48 (0.41–0.57); <0.001		0.43 (0.34–0.54); <0.001	
ORR N (%)	5 (1)	1 (0.4)	6 (4)	0 (0)	8 (1.6)	1 (0.4)	1 (1.1)	0 (0)
*p*	0.19		0.05 *		0.29		0.55	

* One-sided. Abbreviations: R: regorafenib; *P*: placebo; *N*: number; mOS: median overall survival; HR: hazard ratio; CI: confidence interval; mPFS: median progression free survival; ORR: objective response rate.

**Table 2:** Ongoing trials of rechallenge with anti-EGFR antibodies.

Study	Phase	Patient Population (*N*)	Line of Treatment	Regimen	Liquid Biopsy Selection	Primary Endpoint
VELO (EudraCT Number 2018-001600-12)	II	112	Third-line treatment	Trifluridine/tipiracil + panitumumab vs. trifluridine/tipiracil	No	PFS
PARERE (EudraCT Number 2019-002834-35)	II	220	Third-line treatment	Panitumumab > regorafenib vs. Regorafenib > panitumumab	Yes	OS
PULSE (NCT03992456)	II	120	≥Third-line treatment	Panitumumab vs. trifluridine/tipiracil or regorafenib	Yes	OS
FIRE-4 (NCT02934529)	II	230	Third-line treatment	Irinotecan + cetuximab vs. Regorafenib or trifluridine/tipiracil	No	OS
A-REPEAT (NCT03311750)	II	33	Third-line treatment	Irinotecan + cetuximab	No	ORR
NCT03524820	II	60	Third-line treatment	Cetuximab	No	ORR
CHRONOS (NCT03227926)	II	27	Third-line treatment	Panitumumab	Yes	ORR
CAPRI 2 GOIM (EudraCT Number 2020-003008-15)	II	200	≥Second-line treatment	Second-line therapy: FOLFOX + cetuximab vs. FOLFOX + bevacizumab Third-line therapy: Irinotecan plus cetuximab vs. Regorafenib or trifluridine/tipiracil	Yes	NA
CAVE (EudraCT Number 2017-004392-32)	II	75	Third-line treatment	Cetuximab + avelumab	No	OS
NCT03087071 (cohort 3)	II	84	Third-line treatment	Panitumumab	Yes	ORR

Abbreviations: *N*: number; PFS: progression-free survival; OS: overall survival; ORR: objective response rate; NA: not available.

**Table 3:** Main anti-angiogenic MKIs tested as single agents in chemorefractory mCRC.

Agent	Targets	Study Design	Main Eligibility Criteria	Primary Endpoint	Results
Fruquintinib [99]	VEGFR1-3	Phase III trial (vs. placebo)	≥2 prior lines Prior anti-VEGF (not anti-VEGFR) allowed	OS	HR 0.55; *p* 0.00016
Anlotinib [101]	VEGFR2, FGFR-1, PDGFRβ	Phase III trial (vs. placebo)	≥2 prior lines Prior anti-angiogenic allowed	OS	HR 1.02; *p* 0.87
Apatinib [102]	VEGFR-2	Non-randomized phase II trial	≥2 prior lines Prior anti-angiogenic allowed	PFS	4.8 months
Cabozantinib [103]	VEGFR2, MET, RET, KIT, AXL, FLT3	Non-randomized phase II trial	Patients refractory to fluoropyrimidine, irinotecan, oxaliplatin, and bevacizumab	12-week PFS rate	34%
Nintedanib [104]	VEGFR1-3, PDGFRα-β, FGFR1-3, RET, FLT3	Phase III trial (vs. placebo)	Patients refractory to fluoropyrimidine, irinotecan, oxaliplatin, bevacizumab (or aflibercept)	OS, PFS	HR 1.01; *p* 0.87 HR 0.58; *p* < 0.0001
Famitinib [105]	VEGFR2, PDGFRβ, KIT	Phase II trial (vs. placebo)	≥2 prior lines Prior antiangiogenic allowed	PFS	HR 0.60; *p* 0.004

Abbreviations: MKIs: multi-kinase inhibitors; mCRC: metastatic colorectal cancer; OS: overall survival; HR: hazard ratio; PFS: progression free survival.
